# Natural History of Anal Dysplasia in an HIV-Infected Clinical Care Cohort: Estimates Using Multi-State Markov Modeling

**DOI:** 10.1371/journal.pone.0104116

**Published:** 2014-08-07

**Authors:** William C. Mathews, Wollelaw Agmas, Edward R. Cachay, Bard C. Cosman, Christopher Jackson

**Affiliations:** 1 Department of Medicine, University of California San Diego, San Diego, California, United States of America; 2 Department of Surgery, University of California San Diego, San Diego, California, United States of America; 3 Medical Research Council Biostatistics Unit, Cambridge Institute of Public Health, Cambridge, United Kingdom; Technische Universität Dresden, Medical Faculty, Germany

## Abstract

**Objectives:**

(1) To model the natural history of anal neoplasia in HIV-infected patients using a 3-state Markov model of anal cancer pathogenesis, adjusting for cytology misclassification; and (2) to estimate the effects of selected time-varying covariates on transition probabilities.

**Design:**

A retrospective cytology-based inception screening cohort of HIV-infected adults was analyzed using a 3-state Markov model of clinical pathogenesis of anal neoplasia.

**Methods:**

Longitudinally ascertained cytology categories were adjusted for misclassification using estimates of cytology accuracy derived from the study cohort. Time-varying covariate effects were estimated as hazard ratios.

**Results:**

(1) There was a moderate to high probability of regression of the high grade squamous intraepithelial lesion (HSIL) state (27–62%) at 2 years after initial cytology screening; (2) the probability of developing invasive anal cancer (IAC) during the first 2 years after a baseline HSIL cytology is low (1.9–2.8%); (3) infrared coagulation (IRC) ablation of HSIL lesions is associated with a 2.2–4.2 fold increased probability of regression to <HSIL; and (4) antiretroviral therapy, suppressed HIV plasma viral load, and CD4 ≥350/mm^3^ are each associated with reduced probability of progression from <HSIL to HSIL.

**Conclusions:**

The finding of moderate to high rates of regression of the HSIL state accompanied by low rates of progression to IAC should inform both screening and precursor treatment guideline development. There appears to be a consistent and robust beneficial effect of antiretroviral therapy, suppressed viral load, and higher CD4 on the transition from the <HSIL state to the HSIL state.

## Introduction

Understanding the natural history and clinical pathogenesis of anal neoplasia in HIV-infected patients requires both the availability of longitudinal datasets that capture key transitions and endpoints as well as analytic methods that take into account the dynamic nature of the process, involving the possibility of progression and regression among states of precursors to invasive anal carcinoma under the influence of identifiable prognostic factors. We had access to the longitudinal experience of an inception cohort of HIV-infected adults systematically screened for invasive anal cancer and its precursor states. The specific aims of this study were: (1) to model the natural history of anal neoplasia in HIV-infected patients by estimating cytology-based transition probabilities and transition rates (per person-year) under a 3-state model of anal cancer pathogenesis, adjusting for cytology misclassification; and (2) to estimate the effects of selected time-varying covariates on transition probabilities: CD4+ lymphocyte category, HIV plasma viral load suppression, antiretroviral therapy, smoking, and treatment of anal cancer precursors with infrared coagulation (IRC).

## Methods

In 2001, the University of California San Diego (UCSD) Owen Clinic implemented systematic screening for anal cancer and its precursors in all HIV-infected patients under care. The screening algorithm included baseline anal cytology and digital rectal examination (DRE) for all patients under care followed by triage to high resolution anoscopy (HRA) evaluation in patients with either anorectal signs or symptoms or abnormal screening cytology defined as the following Bethesda 2001 cytology diagnostic categories: atypical squamous cells of uncertain significance (ASCUS), low grade intraepithelial lesion (LSIL), atypical squamous cells can't exclude high grade (ASC-H), and high grade intraepithelial lesion (HSIL) [1]. Anal cytology was obtained using a moistened Dacron swab and processed using conventional slide fixation between 2001–2006 and using SurePath liquid cytology media thereafter. HRA with biopsy was performed by trained clinicians using methods previously described [2]. Followup screening with cytology and DRE was recommended annually by clinic protocol for all patients. Because of limited availability of HRA-qualified clinicians, patients with HSIL cytology or palpable abnormalities were preferentially triaged to HRA. Prior to 2007, treatment for anal cancer precursors was not offered. Starting in 2007, patients with HSIL lesions identified at HRA were offered the option of ablative treatment using IRC. An evaluation of this screening program, prior to offering IRC ablation, has been published [3].

We conducted a retrospective inception cohort analysis of HIV-infected patients under care at the UCSD Owen Clinic between 2001-2012. Patients were eligible for cohort analysis if they had: (1) confirmed HIV-infection; and (2) availability of at least 2 longitudinally obtained cytology results (in the absence of a diagnosis of invasive anal cancer [IAC]) or at least one cytology result followed by a diagnosis of IAC. Patients were excluded from cohort analysis if they had: (1) a diagnosis of IAC prior to screening program entry; (2) treatment for anal cancer precursors prior to screening program entry.

For each patient, follow-up time began on the date of the first anal cytology and ended on the first of either the date of IAC diagnosis or the date of the last anal cytology in the study period. IAC diagnosis was ascertained by linking the diagnostic database of the clinic electronic medical record to the UCSD Cancer Registry and validated by review of histopathology reports.

**Figure 1:**
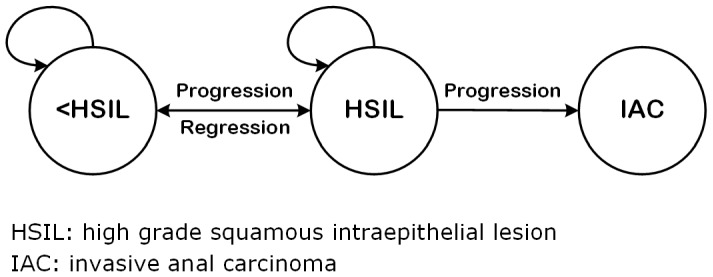
Three State Markov Model of Clinical Pathogenesis of Anal Neoplasia.

We defined a 3-state Markov model of IAC pathogenesis assuming that HSIL is the immediate precursor lesion to invasive anal cancer [4], [5] from which both progression to IAC and regression to less than HSIL (<HSIL) may occur (Figure 1). IAC is considered an absorbing state from which regression does not occur in the absence of definitive treatment. The model state <*HSIL* included the cytologic diagnoses: no atypical or malignant cells, atypical squamous cells of uncertain significance (ASCUS), and low grade squamous intraepithelial lesion (LSIL). The *HSIL* state included the cytology diagnoses: atypical squamous cells, can't rule out high grade (ASC-H) and high grade squamous intraepithelial lesion (HSIL). Results reported as unsatisfactory or squamous intraepithelial lesion (SIL) not otherwise specified were excluded. Cytologic diagnoses were taken as reported by the UCSD clinical cytopathology laboratory.

The model expresses how patients move between states in continuous time. At any time, a patient may progress to a more severe state, or regress to a less severe state as illustrated by the constraints in Figure 1. This model relies on the standard Markov assumption that a patient's risk of transition to another health state depends only on the health state at the current time [6]. The model also assumes that transition intensities or rates remain constant over time, after adjusting for time-varying covariates which are assumed to be constant between the times they are observed.

We report estimates of both transition intensities (which may be interpreted as transition rates per person per year, or hazards) as well as 2 and 5 year transition probabilities. Since transition intensities are assumed piecewise-constant over time, they may be used to estimate a matrix of transition probabilities between baseline and any future time whose (*r,s*) entry is defined as the probability that a patient occupies state *s* at time *t* given they are in state *r* at baseline. It should be noted, however, that a transition probability (with the exception of the transition from HSIL to IAC which is an absorbing state) should not be interpreted as a cumulative incidence. For example, a patient who transitions from HSIL at time 0 to <HSIL at time 1 year may have progressed and regressed between <HSIL and HSIL any number of times in this one-year period. However, this is rare in practice.

In order to maintain an inception screening cohort, the model is cytology based to avoid the selection bias conferred by restricting analysis to patients who were triaged to undergo HRA with biopsy because of antecedent HSIL cytology or abnormal DRE. It is known, however, that both cytology and HRA-directed punch biopsy are imperfect indicators of true histopathologic severity in the anal canal [7], [8]. We therefore modeled state transitions using continuous time hidden Markov models, using the R package *msm* [9], [10], that account for misclassification due to the limitations of both cytology and HRA-directed punch biopsy. Two cytology misclassification matrices were defined using results of previous research from the Owen Clinic anal dysplasia cohort [8]. We first modeled cytology misclassification assuming a sensitivity (SE [95% C.I.] = 0.66 [0.50–0.81]) and specificity (SP [95% C.I.] = 0.90 [0.85–0.95]) of cytology against HRA-directed punch biopsy considered as a gold standard (measured without error). We then modeled cytology misclassification accounting for the fallibility of HRA-directed punch biopsy as a reference standard (SE [95% C.I.]  = 0.89 [0.78–0.95], SP [95% C.I.]  = 0.96 [0.92–0.98]), based on extrapolation from the measured sensitivity and specificity of cervical punch biopsy (with large loop excision of the cervical transformation zone [LLETZ] histopathology as the reference standard [11]) and on the assumption of conditional independence of cytology and biopsy results given true disease status. Consistent with recent recommendations for a unified histopathologic nomenclature for all HPV-associated preinvasive squamous lesions of the lower anogenital tract [12], we use the term HSIL (in the context of our own analysis) to refer to cytology misclassification-adjusted estimates of the true histopathologic state of the anal canal including the diagnostic categories anal intraepithelial neoplasia (AIN) 2 and 3. Model goodness-of-fit was evaluated by graphically comparing observed to expected state prevalences over a two year period from the initial cytologic examination.

In order to avoid a form of prevalence-incidence bias [13], we considered IAC diagnoses within 180 days after the first cytology result to be prevalent cases and excluded them from the primary *IAC_180_ analysis*. Because the width of this IAC exclusion window was arbitrarily selected to take into account delays in referral to HRA and further delays in definitive biopsy of IAC, we performed a sensitivity analysis that reduced the IAC exclusion window to 30 days after the first cytology result (*IAC_30_ analysis*). Final analytic datasets for both *IAC_180_* and *IAC_30_* analyses are available as a Supplementary file (Data Analysis File S1.xlsx).

### Ethics Statement

This research, including the procedure for documenting patient consent, was approved by the UCSD Human Research Protection Program (Project #071931). Written informed consent was obtained from patients to contribute clinical and laboratory data collected during routine care under the UCSD Owen Clinic master protocol.

## Results

During the study period (2001–2012), 2804 patients met eligibility criteria for inclusion in the analysis. Table 1 presents characteristics of the study participants. Median age was 40 and 11% were female. By race/ethnicity, 30% were black or Hispanic. Median CD4+ lymphocyte count was 384 (IQR 217–572) cells/mm^3^. Compared to demographic characteristics of the entire clinic population, study participants were more likely to be white (62% vs. 53%, respectively) and men having sex with men (MSM) who were not injection drug users (IDU) (78% vs. 62%, respectively). Three quarters of study participants were on antiretroviral therapy of whom 64% had a HIV plasma viral load ≤400 copies/mm^3^. Thirty percent were smokers at study entry. Patients were followed for a median of 4.0 years (IQR 2.0–7.1) and underwent a median (IQR) of 5 (3–8) longitudinally collected cytology examinations. Per year of patient followup, the median (IQR) number of cytologies per patient was 1.1 (0.8–1.5). Eight percent (n = 218) underwent one or more IRC ablations for HSIL lesions between 2007–2012. Comparing IRC recipients to non-recipients, there were no differences by age, race/ethnicity, initial CD4 category (≥350 vs.<350 cells/mm^3^), initial HIV viral load (≥400 vs.>400 copies/mm^3^), or smoking status. IRC recipients were more likely than non-recipients to be male (95% vs. 88%, p = 0.002) and to have HSIL as their first cytology result (28% vs. 13%, p<0.0001). The median number of HSIL cytology results was also greater among IRC recipients than non-recipients (median [IQR]: 2 [1]–[4] vs. 0 [0–1], Wilcoxon rank-sum p<0.0001).

**Table 1:** Study Participant Characteristics (n = 2804).

Characteristic		n	Percent
**Age at entry (years Median (IQR))**		40.2	(34.1,46.4)
**Sex**	Female	310	11
	Male	2494	89
**Race/Ethnicity**	White	1730	62
	Hispanic	490	17
	Black	357	13
	Other/Unknown	227	8
**HIV Risk Factor**	MSM_1_	2180	78
	IDU_2_ (not MSM)	133	5
	Heterosexual (not IDU)	367	13
	Other/Unknown	122	4
**Baseline CD4**	<350/mm^3^	1230	44
	≥350/mm^3^	1551	55
	missing	23	1
**Baseline HIV viral load**	≤400 copies/mm^3^	1365	49
	>400 copies/mm^3^	1412	50
	missing	27	1
**On antiretroviral therapy**	Yes	2807	75
	No	707	25
**Smoking**	Yes	837	30
	No	1967	70
**Baseline cytology result_3_**	NAMC	829	29
	ASCUS	919	33
	LSIL	663	24
	ASC-H	76	3
	HSIL	317	11
**≥1 IRC_4_ after first cytology**	Yes	218	8
	No	2586	92
**No. cytology results/patient**	Median (IQR)	5	(3,8)

1. MSM: men having sex with men.

2. IDU: injection drug use.

3. NAMC: no atypical or malignant cells; ASCUS: atypical squamous cells of uncertain significance; LSIL: low grade squamous intraepithelial lesion; ASC-H: atypical squamous cells, can't exclude high grade; HSIL: high grade squamous intraepithelial lesion.

4. IRC: infrared coagulation.

### IAC Ascertainment

Thirty five patients were diagnosed with IAC on or after the first cytology date. Of these, 23 were diagnosed with IAC more than 180 days after the first cytology result and were included in the *IAC_180_ analysis*. An additional 10 IAC diagnoses were made between 30–180 days after the first cytology and these were included in the *IAC_30_ analysis* (total IAC_30_ n = 33). Confirmatory biopsy reports were available for review in 96% of IAC cases.

### Markov Model Estimates of 2 and 5-year Transition Probabilities and Person-Time Rates

Because we found that the time-updated use of IRC was a significant predictor of the regression of HSIL to less than HSIL states (see below) and because our first study aim was to approximate estimates of the natural history of AIN, we present estimates of transition probabilities standardized to the IRC-unexposed category (Table 2). The 2-year transition probability of progression from less than HSIL to HSIL varied from 7–12 percent while the probability of regression of HSIL varied from 27–62 percent depending on cytology misclassification assumptions. The wide range of HSIL regression estimates was determined by cytology misclassification assumptions, with the higher estimate (62%) associated with the correction of cytology sensitivity and specificity for the measurement error of the reference standard punch biopsy. The probability of progression from HSIL to IAC varied from 1.3% to 2.8% at 2 years and from 2.1% to 5.6% at 5 years. The range of these estimates was determined more by the width of the IAC exclusion window (≤180, ≤30 days) than by cytology misclassification assumptions. Examination of model goodness-of-fit plots showed close approximation between observed and expected state prevalence estimates through a 5 year modeling timeframe.

**Table 2:** Estimated 2 and 5 Year Transition Probabilities (95% C.I) for Progression and Regression in 3-State Hidden Markov Model Adjusted for Cytology Misclassification Assumptions, by IAC-Exclusion Window (≤180 vs.≤30 days)_1_.

			Transition from <HSIL		Transition from HSIL_3_		
IAC exclusion window_2_	Cytology Misclassification Assumptions (Sensitivity / Specificity)	Timeframe of Transition Probability Estimates	To <HSIL	To HSIL	To <HSIL	To HSIL	To IAC
**≤180 days**	**0.66/0.90**	**2 years**	0.93 (0.92–0.94)	0.07 (0.06–0.08)	0.28 (0.22–0.34)	0.71 (0.64–0.76)	0.019 (0.013–0.029)
	**0.66/0.90**	**5 years**	0.87 (0.84–0.88)	0.13 (0.11–0.15)	0.51 (0.43–0.59)	0.45 (0.37–0.53)	0.038 (0.025–0.059)
	**0.89/0.96**	**2 years**	0.88 (0.87–0.89)	0.12 (0.11–0.13)	0.62 (0.58–0.66)	0.37 (0.32–0.41)	0.013 (0.008–0.021)
	**0.89/0.96**	**5 years**	0.84 (0.83–0.85)	0.15 (0.14–0.17)	0.80 (0.78–0.82)	0.18 (0.17–0.20)	0.021 (0.014–0.032)
**≤30 days**	**0.66/0.90**	**2 years**	0.93 (0.92–0.94)	0.07 (0.06–0.08)	0.27 (0.22–0.34)	0.70 (0.63–0.75)	0.028 (0.020–0.040)
	**0.66/0.90**	**5 years**	0.87 (0.84–0.89)	0.13 (0.11–0.15)	0.51 (0.43–0.59)	0.44 (0.35–0.51)	0.056 (0.039–0.079)
	**0.89/0.96**	**2 years**	0.88 (0.87–0.89)	0.12 (0.11–0.13)	0.62 (0.58–0.66)	0.36 (0.32–0.40)	0.019 (0.014–0.028)
	**0.89/0.96**	**5 years**	0.84 (0.83–0.85)	0.15 (0.14–0.17)	0.79 (0.77–0.81)	0.18 (0.16–0.20)	0.031 (0.021–0.042)

1. Model estimates fit with time-updated infrared coagulation (IRC) indicator and standardized to reflect transition probabilities for those who never underwent IRC. Thus, these transition probabilities estimate the untreated natural history of AIN.

2. IAC exclusion window: Cases of invasive anal carcinoma (IAC) diagnosed within 180 or 30 days of the first cytology result, respectively, were considered prevalent cases and were therefore excluded from analysis.

3. Transition from HSIL: HSIL: high grade squamous intraepithelial lesion; IAC: invasive anal carcinoma.

Table 3 presents estimates of state transition rates adjusted for cytology misclassification assumptions and stratified by width of the IAC exclusion window. These rates may be interpreted as the risk per person per year of the indicated state transitions, standardized to the experience of those who never underwent IRC ablation. The annual rate of progression from <HSIL to HSIL varied from 0.04 to 0.11 while the annual rate of regression of HSIL varied from 0.17 to 0.58. The rate of progression of HSIL to IAC varied from 0.01 to 0.02.

**Table 3:** Estimates of State Transition Rate (per person-year) adjusted for Cytology Misclassification Assumptions, by IAC-Exclusion Window (≤180 vs. ≤30 days)_1_.

IAC Exclusion Window_2_	Cytology Misclassification Assumptions (Sensitivity / Specificity)	State Transition Rate (per person-year)_3_ (95% CI)		
		<HSIL → HSIL	HSIL → <HSIL	HSIL → IAC
**≤180 days**	**0.66/0.90**	0.04 (0.036–0.054)	0.17 (0.13–0.22)	0.011 (0.007–0.017)
	**0.89/0.96**	0.11 (0.096–0.130)	0.58 (0.50–0.67)	0.011 (0.007–0.016)
**≤30 days**	**0.66/0.90**	0.04 (0.035–0.053)	0.17 (0.13–0.22)	0.017 (0.012–0.024)
	**0.89/0.96**	0.11 (0.096–0.130)	0.58 (0.50–0.67)	0.016 (0.011–0.023)

1. Model estimates fit with time-updated infrared coagulation (IRC) indicator and standardized to reflect transition rates for those who never underwent IRC. Thus, these rates estimate the untreated natural history of AIN if the rates remain constant over time and if the Markov assumption is valid.

2. IAC exclusion window: Cases of invasive anal carcinoma (IAC) diagnosed within 180 or 30 days of the first cytology result, respectively, were considered prevalent cases and were therefore excluded from analysis.

3. State Transitions: HSIL: high grade squamous intraepithelial lesion; IAC: invasive anal carcinoma.

### Covariate effects on State Transition Rates

Table 4 presents unadjusted hazard ratios (HR) for the following time-updated covariates: (1) IRC ablation, (2) antiretroviral therapy, (3) HIV plasma viral load, (4) CD4 category, and (5) smoking. The hazard ratios are estimated separately for cytology misclassification assumptions and for width of IAC exclusion window. As noted above, IRC ablation was strongly associated with regression of HSIL lesions (HR_HSIL→<HSIL_ varying from 2.2 to 4.2). IRC ablation had no statistically significant effect on progression of HSIL to IAC, but this must be interpreted in the context of a small number of observed IAC events.

**Table 4:** Estimated Unadjusted Hazard Ratios (95% CI) of Time-Updated Covariate Effects on State Transitions, by Misclassification Assumptions and by IAC-Exclusion Window (≤180 vs.≤30 days).

Covariate	IAC exclusion window_2_	Cytology Misclassification Assumptions (Sensitivity / Specificity)	<HSIL to HSIL	HSIL to <HSIL	HSIL to IAC_3_
**1. IRC_1_ [reference: no IRC]**	**≤180 days**	**0.66/0.90**	2.2 (0.6–7.9)	4.2 (2.0–8.5)	2.7 (0.6–11.7)
		**0.89/0.96**	2.1 (0.95–4.6)	2.3 (1.3–4.0)	3.2 (0.7–13.6)
	**≤30 days**	**0.66/0.90**	2.2 (0.6–8.0)	4.2 (2.0–8.6)	1.8 (0.4–7.7)
		**0.89/0.96**	2.1 (0.96–4.7)	2.2 (1.3–3.9)	2.1 (0.5–9.0)
**2. ART_4_ [reference: no ART]**	**≤180 days**	**0.66/0.90**	**0.4 (0.2–0.6)**	0.9 (0.4–2.1)	2.2 (0.5–9.4)
		**0.89/0.96**	**0.5 (0.4–0.8)**	0.9 (0.6–1.3)	2.1 (0.5–9.1)
	**≤30 days**	**0.66/0.90**	**0.4 (0.2–0.6)**	0.9 (0.4–2.1)	3.2 (0.8–13.7)
		**0.89/0.96**	**0.5 (0.4–0.7)**	0.8 (0.6–1.3)	3.2 (0.8–13.4)
**3. HIV Viral load [reference: >400 copies/mm^3^]**	**≤180 days**	**0.66/0.90**	**0.3 (0.2–0.5)**	1.3 (0.7–2.3)	1.6 (0.7–3.9)
		**0.89/0.96**	**0.5 (0.4–0.7)**	1.1 (0.8–1.5)	1.6 (0.6–3.8)
	**≤30 days**	**0.66/0.90**	**0.3 (0.2–0.5)**	1.3 (0.7–2.3)	1.2 (0.6–2.5)
		**0.89/0.96**	**0.5 (0.4–0.7)**	1.1 (0.8–1.4)	1.2 (0.6–2.5)
**4. CD4 Category [reference: <350/mm^3^]**	**≤180 days**	**0.66/0.90**	**0.3 (0.2–0.5)**	0.8 (0.5–1.3)	1.4 (0.6–3.3)
		**0.89/0.96**	**0.4 (0.3–0.6)**	0.9 (0.6–1.1)	1.4 (0.6–3.2)
	**≤30 days**	**0.66/0.90**	**0.3 (0.2–0.5)**	0.8 (0.5–1.3)	1.1 (0.5–2.2)
		**0.89/0.96**	**0.4 (0.3–0.6)**	0.8 (0.6–1.1)	1.1 (0.5–2.1)
**5. Smoking [reference: not smoking]**	**≤180 days**	**0.66/0.90**	1.1 (0.7–1.7)	0.8 (0.5–1.4)	1.2 (0.5–2.9)
		**0.89/0.96**	1.0 (0.7–1.4)	0.9 (0.7–1.2)	1.3 (0.6–3.0)
	**≤30 days**	**0.66/0.90**	1.1 (0.7–1.7)	0.9 (0.5–1.4)	1.2 (0.6–2.5)
		**0.89/0.96**	1.0 (0.7–1.4)	0.9 (0.7–1.2)	1.3 (0.6–2.6)

1. IRC: infrared coagulation.

2. IAC exclusion window: Cases of invasive anal carcinoma (IAC) diagnosed within 180 or 30 days of the first cytology result, respectively, were considered prevalent cases and were therefore excluded from analysis.

3. State Transitions: HSIL: high grade squamous intraepithelial lesion; IAC: invasive anal carcinoma.

4. ART: antiretroviral therapy.

Consistent favorable (protective) effects were observed for antiretroviral therapy (HR varying from 0.4 to 0.5), suppressed (≤400 copies/mm^3^) HIV plasma viral load (HR 0.3–0.5), and higher (≥350/mm^3^) CD4+ lymphocyte category (HR 0.3–0.4) on the transition from <HSILto HSIL. But these covariates appeared not to influence regression from HSIL to <HSIL or progression from HSIL to IAC. Somewhat surprisingly, we did not observe effects of smoking on any of the modeled state transitions.

## Discussion

This study provides quantitative estimates of the dynamic natural history of anal intraepithelial neoplasia in a cohort of HIV-infected patients who were systematically screened for AIN as part of routine care. Its inferences are based on longitudinal and repeated cytologic follow-up with careful ascertainment of the endpoint invasive anal carcinoma. A particular strength of our modeling approach is the simultaneous estimation of multiple dynamic state transition parameters rather than focusing only on one endpoint (e.g. IAC incidence). Our principal findings are that: (1) there is a moderate to high probability of regression of HSIL (27–62%) at 2 years after initial cytology screening; (2) the probability of developing IAC during the first 2 years after a baseline HSIL cytology is low (1.9–2.8%); (3) IRC ablation of HSIL lesions is associated with a 2.2–4.2 fold increased probability of regression to <HSIL but not associated with an effect on progression to IAC; and (4) antiretroviral therapy, suppressed HIV plasma viral load, and CD4≥350/mm^3^ are each associated with reduced probability of progression from <HSIL to HSIL with a relative risk reduction of progression in the range of 50–70%. However, there was no discernable effect of these covariates on either regression of HSIL or progression of HSIL to IAC.

### Design-related issues

Interpretation of these findings may be model dependent. Multi-state Markov models have been used to understand the natural history of cervical neoplasia [14]–[17] as well as to model the cost-effectiveness of screening programs for anal cancer and its precursors [18]–[20]. A recent analysis of cervical cytology and human papilloma virus (HPV) DNA samples in a cohort of HIV-infected and high risk HIV-negative women used a 3-state cytology-based Markov model ([1] no SIL, [2] SIL, and [3] the absorbing state of treatment for SIL or invasive cervical cancer) to determine factors associated with transitions between cervical cytopathologic states [21]. In the latter study, SIL was defined, in the primary analysis, to include ASCUS, LSIL, and HSIL cytologic findings. Our model focused on HSIL as the putative necessary precursor lesion to invasive cancer, recognizing that lower degrees of dysplasia may reflect effects of infection with non-oncogenic HPV strains (e.g. HPV types 6 and 10).

We acknowledge that our decision to classify normal, ASCUS, and LSIL cytologies as a single state creates heterogeneity as some patients with normal or ASCUS cytologies may not be HPV-infected while those with LSIL have at least non-oncogenic strain infection. However, epidemiological studies have shown that among HIV-infected MSM, prevalent anal HPV infection is the rule (>80%) with multiple infecting types also extremely common (>60%) [22], [23]. Oncogenic HPV type 16 is the most common infecting strain (∼30%) and tends to have a lower clearance rate and higher mean retention time than low risk HPV types [24]. In addition, incident HPV is common among HIV-infected MSM. For example, de Pokomandy et al. reported a cumulative incidence of HPV-16 infection of 33% at 36 months followup in a cohort of HIV-infected MSM. [22] So while our <HSIL state is heterogeneous, even those with normal baseline cytology are likely to be at risk for either prevalent or incident HPV infection. Moreover, our research interest focuses especially on those with HSIL cytology not only because it is the precursor to IAC but also because current recommendations focus on treatment of HSIL and not on lower grade lesions [25].

A second study design related issue is our decision to model AIN natural history using a cytology-based inception cohort rather than the subset of our cohort that underwent HRA with concurrent biopsy and cytology. To illustrate the selection factor introduced by restricting our analysis to the HRA sub-cohort, the proportion of baseline HSIL cytology among those subsequently referred to HRA (n = 629) was 38% while the comparable proportion among those never referred to HRA (n = 2175) was 7% (p<0.0001). This difference is simply the result of the clinic triage algorithm based on limited HRA availability. The selection bias introduced by conditional referral to HRA will be avoided in prospective study designs that use both cytology and HRA-directed biopsy concurrently and longitudinally in all screened patients. The results of one such natural history study are eagerly awaited [26].

A third design issue concerns our methods of adjusting for cytology misclassification of the true but unknown severity of histopathologic AIN. Cytology is known to lack sensitivity for detection of HSIL [7], but as we have argued elsewhere, HRA-directed punch biopsy is not itself a true gold standard [8]. There is considerable heterogeneity among reports of anal cytology sensitivity and specificity with HRA-directed biopsy as the reference standard and the pooled sensitivity for HSIL on biopsy using a cytology cut-point of (HSIL or ASC-H) vs (normal, ASCUS, or LSIL) was only 30% (95% CI: 19–44%) in a recent meta-analysis [27]. We elected to apply the sensitivity (66%) and specificity (90%) estimated in our own cohort to correct our state transition estimates for misclassification due to the limitations of cytology. We further modeled the implications of taking into account the fallibility of the punch biopsy reference standard using a methodology developed to characterize the accuracy of cervical punch biopsy against a reference standard from LLETZ histopathology [11], [28]. Both sets of cytology sensitivity and specificity modeling assumptions were based on point estimates and do not take into account imprecision due to sampling variability as reflected in the width of their respective confidence intervals (see Results). Some external validation of our misclassification-adjusted cytology-based methodology may be seen when examining estimates of progression to and spontaneous regression from HSIL in an independent longitudinal cohort including both HIV-infected (73%) and uninfected (27%) patients [29]. In this Australian cohort report, the state of tissue histopathology was defined as a composite of the highest grade of cytology or biopsy; and of 419 patients with baseline cytology, 183 (44%) had biopsy results. The rate of high grade AIN regression among the HIV-infected patients was 19.2 per 100 person-years (p-yrs), which is very close to our estimate regression rate of 17 per 100 p-yrs assuming a sensitivity and specificity of cytology of 0.66 and 0.90, respectively. For the rate of progression from <HSIL to HSIL, Tong reported a rate among the HIV-infected of 6.6 per 100 p-years while our estimate for the same transition was 4 per 100 p-yrs applying the same cytology misclassification assumption (Table 3). Estimates in our work based on adjustment for the fallibility of the punch biopsy as reference standard cannot be directly compared to those in the Tong paper, which did not make a comparable adjustment. Lacking external validation of estimates based on adjustment for the fallibility of the punch biopsy as reference standard, such estimates should be taken with caution.

### Estimates of progression to IAC

Risk and rates of progression from HSIL to IAC among HIV-infected patients who were not treated for HSIL is imprecisely known. In a recent meta-analysis, Machalek et al. [30] proposed a *theoretical* progression rate from high grade AIN to IAC among HIV-infected men in the HAART era of one in 377 per year in the absence of treatment for precursor lesions. The few available longitudinal estimates of the risk and rates of progression from HSIL to IAC have been recently reviewed [31]. More recently, Dalla Pria et al. reported on the experience of an HIV-positive MSM cohort in which HRA with intervention for HSIL was routinely offered [32]. In this HSIL-treated cohort, the estimated rate of IAC from first histopathologic diagnosis of high grade AIN (including AIN-2 and AIN-3) ascertained at the first HRA was 6.1 per 1000 person-years (95% CI: 4.2–7.8); this rate corresponds to a per person per year rate of 1/164. In comparing estimated rates of progression from HSIL to IAC, several factors related to the precise definition of HSIL in its modeling context need to be taken into account: (1) whether the estimates are based on HSIL diagnosed at first screening procedure or based on HSIL diagnosed at baseline or any subsequent screening event; (2) whether the estimates are based on HSIL diagnosed only by cytology, only by biopsy, by some combination of cytology and biopsy, or by misclassification adjusted cytology and biopsy; (3) whether the estimates are based on baseline HSIL or time updated HSIL; and (4) what modeling approach is used (e.g. multi-state modeling, regression modeling). To illustrate the impact of these factors on HSIL progression estimates, we refer to 2 different estimates derived from the same cytology-based dataset modeled in this paper. We estimated using Cox regression analysis the rate of HSIL progression to IAC, ascertained from the time of the HSIL cytology at baseline screening (unadjusted for misclassification and including data from the 8% of patients who underwent IRC ablation), as one in 263 per year (95% CI: 1/714–1/222 [Cachay et al., 2014 (submitted)]. In contrast, using Markov modeling adjusting for cytology misclassification and standardizing to the experience of patients never exposed to IRC ablation, we estimated the same progression rate to vary from 1/59 (0.017) to 1/94 (0.011) per person per year depending on the width of the IAC exclusion window and cytology misclassification assumptions (Table 3). Clearly it is essential to specify not only the study design but also the modeling approach when citing HSIL progression rates.

### Covariate effects

A 2012 Cochrane review of interventions for AIN identified only one randomized controlled trial, of the immunomodulator imiquimod, that met inclusion criteria and concluded that there was no reliable evidence for efficacy of any of the examined interventions [33]. Nonetheless, a number of observational studies have suggested that IRC ablation of HSIL in HIV-infected patients is accompanied by frequent recurrence requiring re-treatment, while the probability of cure of individual HSIL lesions is high [34]–[41]. In the absence of randomized controlled trials, it is not clear that IRC ablation reduces progression to IAC [42]. Our study confirms a substantial IRC treatment effect (2.2–4.2 fold) in downgrading HSIL to less than HSIL. However, no statistically significant effect was observed on the transition probability from HSIL to IAC.

The effect of highly active antiretroviral therapy (HAART) on the natural history of AIN and the incidence of IAC in HIV-infected populations has been recently reviewed [31], [43]. It is clear that the incidence of IAC has increased in the HAART era [44]–[46]. Among published studies that have suggested a beneficial effect of antiretroviral therapy on the natural history of AIN, two were cross-sectional [47], [48] and two longitudinal [49], [50]. Among the cross-sectional studies (both using HRA-directed biopsy), Wilkin et al. found that current antiretroviral therapy was associated with lower risk of any degree of AIN while Van der Snoek et al. found that use of HAART of any duration was associated with lower risk of any degree of AIN. Among the longitudinal studies, de Pokomandy et al. found that HAART use for >4 years was associated with a marginally significant protective effect (OR, 0.28 [95% CI, 0.07–1.06]) on progression from <AIN 2 to AIN 2/3 assessed by HRA while Chiao et al., analyzing the Veterans Administration HIV Clinical Case Registry, found higher proportion of followup time with undetectable HIV plasma viral load predicted lower incidence of IAC. Our study had the strength of simultaneously examining longitudinally assessed state transitions in the *clinical pathogenesis of IAC* and found a strong protective effect for time-updated HAART use (50–60% relative risk reduction) not for the transition of HSIL to IAC or for regression from HSIL to <HSIL, but rather for progression from <HSIL to HSIL. This finding was further supported by our finding that suppressed HIV plasma viral load and CD4≥350, both mediating at least a major part of the benefit of antiretroviral therapy, also reduced the transition probability from <HSIL to HSIL. These findings may be important to correlate with evolving understanding of the molecular pathogenesis of IAC, which seems to involve sequential changes requiring persistence of oncogenic HPV infection, DNA integration, and subsequent genetic changes that may be irreversible [51]–[53].

Smoking is an established cofactor in cervical carcinogenesis and may act at several steps in the causal pathway leading to invasive cervical carcinoma [54], [55]. Studies have identified smoking as a risk factor for IAC [56] and for AIN 2+ histopathology [57] among HIV-infected patients. Our failure to detect a smoking effect on any of the modeled state transitions may be attributable to ascertainment and misclassification biases since we relied on electronic medical record diagnoses of smoking rather than on systematic patient survey of smoking behaviors.

A number of potential data and design limitations of our analysis have been discussed above, including specification of a 3-state model, the use of cytology-based state ascertainment, the impact of cytology misclassification assumptions on model estimates, and the modeling dependency of estimates of progression to IAC. In addition, the absence of HPV-DNA screening limited our ability to identify sub-cohort members most at risk for progression to HSIL and IAC. Lastly, because of the relatively small number of IAC endpoints in our cohort, power to detect covariate effects on the transition from HSIL to IAC is limited.

## Conclusions

Analysis of this longitudinal cytology-based misclassification-adjusted inception screening cohort has yielded simultaneous quantitative state transition estimates that further elucidate the clinical pathogenesis of anal neoplasia in HIV-infected patients. Of particular importance for development of screening and precursor treatment guidelines is the finding of moderate to high rates of spontaneous regression of HSIL lesions in association with much lower rates of progression to invasive anal cancer. This work has added to evolving understanding of the effects of antiretroviral therapy on the natural history of anal neoplasia by identifying potent preventive effects of HAART, suppressed HIV viral load, and higher CD4+ lymphocyte category on the transition from <HSIL to HSIL. Finally, this analysis supports the effectiveness of infrared coagulation ablation of HSIL in downgrading HSIL but detected no effect on progression to invasive cancer.

## Supporting Information

Data Analysis File S1 (XLSX)
